# Research Progress of La_1-x_Sr_x_MnO_3_-Based Flexible Wearable Sensors

**DOI:** 10.3390/mi17050629

**Published:** 2026-05-21

**Authors:** Xiaoqing Xing, Xinjie Fan, Ruoshi Li, Boxin Lu, Yin Ma, Chun Jia, Dong Gao, Jie Wu, Guogang Ren, Mian Zhong

**Affiliations:** 1 College of Aviation Electronic and Electrical Engineering, Civil Aviation Flight University of China, Guanghan 618307, China; xingxiaoqing@cafuc.edu.cn (X.X.); 13540235506@163.com (R.L.); momaqj@163.com (Y.M.); j18096397820@163.com (C.J.); gaodong1303@163.com (D.G.); 2 College of Safety Science and Engineering, Civil Aviation University of China, Tianjin 300300, China; 3 College of Aviation Engineering, Civil Aviation Flight University of China, Guanghan 618307, China; xinjiefan@yeah.net (X.F.); 13394546506@163.com (B.L.); 4 College of Art and Physical Education, Kyungil University, Gyeongsan-si 38428, Republic of Korea; 5 School of Physics, Engineering and Computer Science, University of Hertfordshire, Hatfield AL10 9AB, UK; g.g.ren@herts.ac.uk

**Keywords:** La_1-x_Sr_x_MnO_3_, flexible sensor, wearable device, fabrication process, multimodal sensing

## Abstract

With the rapid development of flexible electronics technology, flexible wearable sensors based on Lanthanum Strontium Manganese Oxide (La_1-x_Sr_x_MnO_3_) have garnered extensive attention in recent years due to their excellent multi-functional integration, environmental stability and biocompatibility. This review systematically analyzes the preparation methods, process optimization strategies, multi-performance integration technologies, and the expansion of the application field of La_1-x_Sr_x_MnO_3_-based flexible sensors. Firstly, the basic characteristics and sensing mechanism of the La_1-x_Sr_x_MnO_3_ material were presented, including its temperature sensitivity, strain response characteristics, and magnetoresistance effect. Secondly, the fabrication process of flexible sensors was elaborately discussed, with a focus on analyzing crucial technologies, such as laser induction and transfer printing technology. Subsequently, the strategies for regulating the electrical, thermal, and mechanical properties of materials through element doping, along with the multimodal sensing integration and signal decoupling methods, were expounded. Furthermore, the actual performance of this type of sensor in fields such as health monitoring, human–computer interaction, and extreme environment applications was summarized. Finally, the challenges and future development directions of La_1-x_Sr_x_MnO_3_-based flexible sensors are outlined, providing theoretical references for the design and optimization of next-generation flexible electronic devices.

## 1. Introduction

With the development of artificial intelligence and the growth of personalized health management demands, flexible electronic devices, which serve as essential information acquisition tools, have demonstrated significant potential in accurate measurement on complex and deformable surfaces [1]. In comparison with traditional rigid electronic devices, flexible devices possess advantages such as low cost, light weight, and conformal integration, overcoming the limitations of traditional devices [2]. They have also performed well in areas such as aircraft structural health monitoring, human motion detection, and wearable health monitoring [3]. Among numerous flexible sensing materials, perovskite manganese oxide, La_1-x_Sr_x_MnO_3_ (LSMO), has become a research focus due to its outstanding electro–thermal performance, excellent mechanical flexibility, and abundant physical effects [4]. The La_1-x_Sr_x_MnO_3_ material exhibits metallic conductivity at room-temperature and features a negative temperature coefficient (NTC) characteristic, which makes it highly suitable for application in the temperature-sensing field. More importantly, by adjusting the doping concentration of Sr^2+^, the electrical and magnetic properties of La_1-x_Sr_x_MnO_3_ can be precisely regulated, optimizing its sensing characteristics [3].

In recent years, researchers have successfully developed a variety of high-performance flexible sensors [5], breaking through the limitations of traditional flexible sensors in room-temperature environments and achieving precise detection within a wider temperature range, which demonstrates excellent environmental adaptability [6]. In the field of health monitoring, flexible wearable respiratory monitoring sensors based on flexible La_0.7_Sr_0.3_MnO_3_/mica films exhibit excellent mechanical bending robustness and multimodal response capabilities to temperature changes, indicating potential for real-time, continuous, and long-term monitoring of dynamic respiratory processes [2]. In the aspect of strain sensing, a La_0.7_Sr_0.3_MnO_3_/mica heterostructure flexible sensor integrates high mechanical bending sensitivity with excellent bending durability (up to 3600 cycles), and also exhibits a sensitive response to external magnetic fields, indicating intrinsic multimodal perception capabilities. Notably, this sensor can operate stably within an extremely wide temperature range of 20 K to 773 K, providing a novel technical solution for flexible electronic applications in extreme environments.

**Figure 1:**
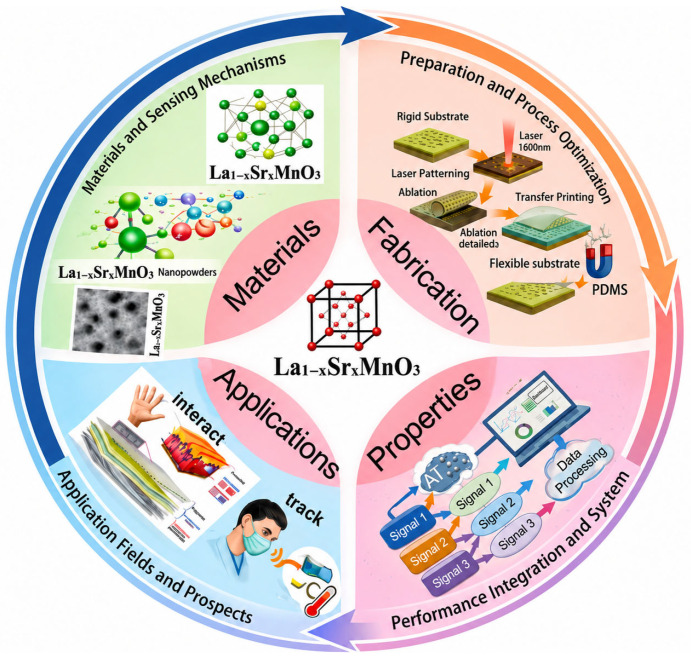
Comprehensive schematic diagram of La_1-x_Sr_x_MnO_3_-based materials.

This review is intended to systematically summarize the most recent research progress of La_1-x_Sr_x_MnO_3_-based flexible wearable sensors, as shown in Figure 1. It focuses on analyzing their preparation methods, process optimization strategies, multi-performance integration technologies, and expansion of the application field, providing a comprehensive reference for researchers in related fields.

## 2. Basic Characteristics and Sensing Mechanism of La_1-x_Sr_x_MnO_3_

### 2.1. Structure and Physical Properties of La_1-x_Sr_x_MnO_3_

**Figure 2:**
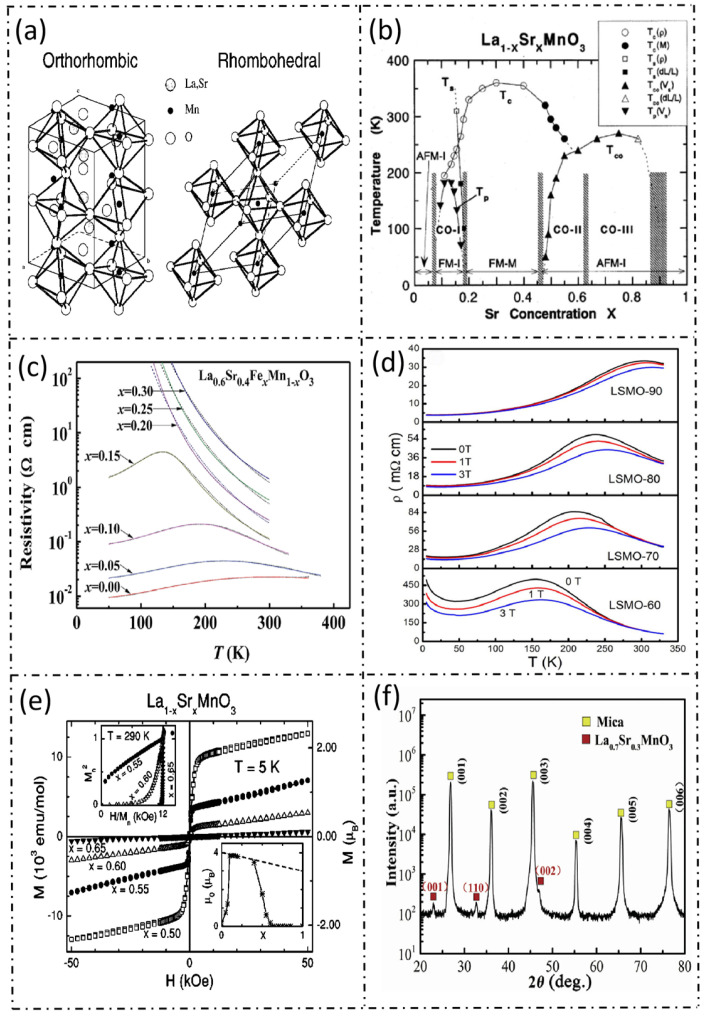
Perovskite structure and key physical properties of La_1-x_Sr_x_MnO_3_. (**a**) Crystal structure of La_1-x_Sr_x_MnO_3_ [1]; (**b**) magnetic phase transition of La1-xSr_x_MnO_3_ [2]; (**c**) electrical conductivity characteristics of La_0.6_Sr_0.4_Fe_x_Mn_1-x_O_3_ [3]; (**d**) magnetic response of La_1-x_Sr_x_MnO_3_ [4]; (**e**) M-H curves of La_1-x_Sr_x_MnO_3_ [5]; (**f**) X-ray diffraction pattern of La_1-x_Sr_x_MnO_3_ [6].

La_1-x_Sr_x_MnO_3_ is a perovskite-structured oxide, with a crystal structure of the ABO_3_ type, as shown in the perovskite structure schematic diagram in Figure 2. In this structure, La^3+^ and Sr^2+^ jointly occupy the A site, and Mn^3+^/Mn^4+^ occupy the B site [3]. This unique crystal structure endows the material with a variety of physical effects, including colossal magnetoresistance (CMR), metal-insulator transition, and charge ordering, etc. The existing research indicates that La_1-x_Sr_x_MnO_3_-based sensors can operate stably within an extremely wide temperature range from 20 K to 773 K, which far exceeds the temperature adaptability range of traditional flexible sensors [2,3].

The electrothermal properties of La_1-x_Sr_x_MnO_3_ materials originate from their distinctive electronic conduction mechanism [3]. In the low-temperature region, La_1-x_Sr_x_MnO_3_ exists in a ferromagnetic metallic state, and its resistivity increases slowly with the increase in temperature, reaching a peak at the Curie temperature. Once it transitions into a paramagnetic insulating state, the resistivity decreases. Consequently, by adjusting the doping concentration of Sr^2+^, the transition temperature corresponding to the resistivity inflection point can be regulated to fulfill the requirements of various application scenarios [3].The main physical properties and corresponding application relevance of La_1-x_Sr_x_MnO_3_ materials are summarized in Table 1.

### 2.2. Analysis of Sensing Mechanism

The multifunctionality of La_1-x_Sr_x_MnO_3_-based flexible sensors is derived from the synergistic effects of various physical phenomena [7]. In terms of temperature sensing, it mainly depends on the thermosensitive properties of the materials [3]. When the sensor is exposed to a temperature-changing environment, the resistance value of the La_1-x_Sr_x_MnO_3_ film will change correspondingly. The temperature field distribution under different breathing states demonstrates significant differences, which enable the discrimination of breathing frequency and patterns [2]. In strain sensing, the piezoresistive effect of the La_1-x_Sr_x_MnO_3_ film plays a dominant role. When mechanical deformation occurs, the stress generated inside the La_1-x_Sr_x_MnO_3_ film will result in lattice distortion and alterations in the energy band structure, thus leading to resistance changes [3]. Additionally, the La_1-x_Sr_x_MnO_3_ material also exhibits a sensitive response to magnetic fields. When an external magnetic field is present, the resistance of La_1-x_Sr_x_MnO_3_ will change due to the magnetoresistance effect [8]. The multi-modal sensor based on magnetic–thermal strain can simultaneously monitor multiple physical parameters, significantly reducing the complexity of wearable devices [5,6,9].

**Figure 3:**
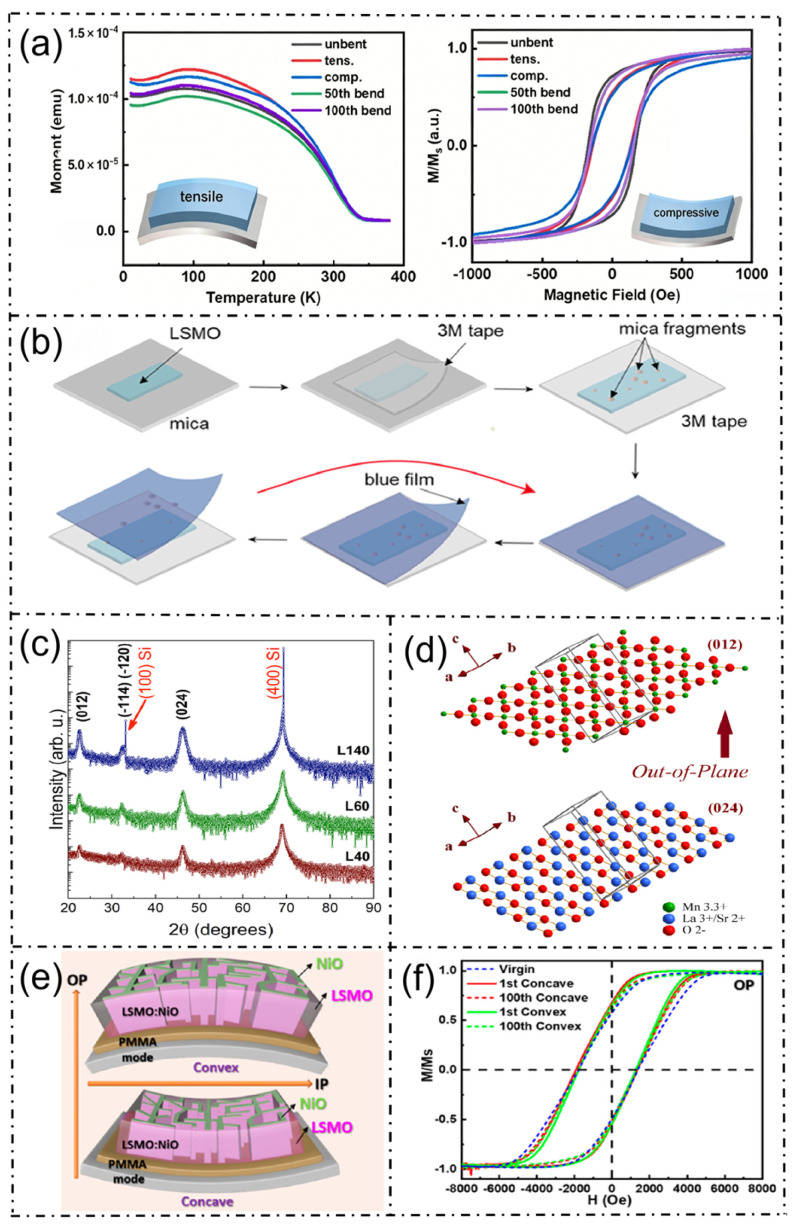
Structural and physical properties of La_1-x_Sr_x_MnO_3_ sensors: Magnetism, electrical resistance, and temperature response. (**a**) Magnetization and magnetoresistance response, illustrating the temperature- and magnetic field-dependent behavior of La_1-x_Sr_x_MnO_3_ thin films under various strain states [10]; (**b**) fabrication of flexible sensors, demonstrating the preparation process of the La_1-x_Sr_x_MnO_3_/Mica composite material [10]; (**c**) XRD structural analysis, with XRD patterns of La_1-x_Sr_x_MnO_3_ thin films with different Sr concentrations showing various crystallographic orientations [7]; (**d**) schematic of crystal structure, explaining the (012)/(024) diffraction peaks and the preferred (012) orientation growth observed in the XRD patterns of (**c**) [7]; (**e**) flexible thin film and magnetoresistance response, illustrating the magnetoresistance behavior and structural characteristics under bending [11]; (**f**) magnetic response curves, hysteresis loops and magnetoresistance response of La_1-x_Sr_x_MnO_3_ under different bending levels [11].

These three core sensing mechanisms and their corresponding experimental verification are systematically presented in Figure 3. This figure illustrates the magnetization and magnetoresistance behavior of La_1-x_Sr_x_MnO_3_ thin films under various strain and temperature conditions, the fabrication process of flexible LSMO/mica composite sensors, the crystallographic orientation evolution of films with different Sr concentrations, and the magnetic response characteristics under repeated bending cycles.

## 3. Preparation Method and Performance Optimization

### 3.1. Laser-Induced Processing Technology

Laser-induced processing technology has demonstrated significant advantages in the fabrication of flexible sensors. Firstly, it offers high processing precision. The spot diameter of a laser can be focused to the micrometer or even nanometer scale, enabling the fabrication of electrodes and sensing channels with extremely narrow line widths and other key structures. Secondly, it features wide material compatibility. Laser-induced processing does not depend on specific chemical reagents and can directly process various materials, including metals (e.g., copper and silver), semiconductors (e.g., graphene and carbon nanotubes), and polymers, without the necessity of changing equipment or significantly making process adjustments. Thirdly, it enables selective processing. On the same flexible substrate, only the target area of the sensing material is etched or modified, which simplifies the integration process of heterogeneous materials. Fourthly, it involves fewer process steps. It can accomplish multiple processes, such as patterning, etching, and surface modification, in a single operation and can promptly meet small-batch and customized sensor demands. Femtosecond laser direct-writing technology is also applicable to the micro–nano processing of La_1-x_Sr_x_MnO_3_ sensors. It enables the direct patterning of functional materials on flexible substrates, such as polyimide (PI), fulfilling the requirements of miniaturized sensing units, thereby avoiding complex lithography processes and significantly simplifying the preparation process [12,13].

### 3.2. Transfer Printing Technology

Transfer printing technology presents an efficient fabrication approach for La_1-x_Sr_x_MnO_3_ sensors. This technique is capable of precisely transferring La_1-x_Sr_x_MnO_3_ materials to specific locations on the sensor, thereby ensuring structural accuracy and performance stability. It is compatible with various substrates and functional materials, making it suitable for the preparation of multiple types of La_1-x_Sr_x_MnO_3_ sensors and expanding the application scope of sensors. In comparison with traditional fabrication methods, this technology does not require complex equipment or harsh conditions such as high temperature and high pressure. The operation process is relatively straightforward, enabling rapid fabrication, improving production efficiency, and reducing costs. For instance, a flexible sensor with V-shaped grooves and planar structures is fabricated using microimprinting technology, with polydimethylsiloxane (PDMS) serving as the substrate and encapsulation material. By adjusting the adhesion force of the elastomer PDMS, the stability requirements for long-term pressure-related signal monitoring in skin-contact scenarios are met [14].

### 3.3. Element Doping

Element doping serves as a crucial approach for optimizing the performance of La_1-x_Sr_x_MnO_3_ materials. The electrical, magnetic, and thermal properties of the materials can be precisely controlled by substituting elements at either the A or B positions. In A-site doping, Sr^2+^ is often employed to partially replace La^3+^ to regulate the Mn^3+^/Mn^4+^ ratio [15]. This, in turn, affects the intensity of the double-exchange interaction and subsequently modifies the electrical conductivity and magnetic properties of the material [16]. This mechanism mainly results from the combined contribution of the superexchange interaction (SE) and the double-exchange interaction (DE) [17]. Kramers [18] first proposed the superexchange theory to explain the antiferromagnetic order phenomenon observed in transition-metal oxides such as MnO. Moreover, it was pointed out that the magnetic Mn ions are indirectly coupled through the 2p orbitals of the bridge O^2-^ ion [19]. As a perovskite oxide, the performance of La_1-x_Sr_x_MnO_3_ not only originates from its crystal structure but is also profoundly influenced by its electronic structure. In the undoped parent compound LaMnO_3_, the Mn ion is situated in an oxygen-octahedral crystal field, with 3d orbitals split and an electronic configuration of 3d^4^ [20]. When Sr^2+^ is introduced for A-site doping to form La_1-x_Sr_x_MnO_3_, the Mn ion presents a mixed-valence state of Mn^3+^ and Mn^4+^, corresponding to the hole doping of the Mn3d band [21]. The magnetic properties of La_1-x_Sr_x_MnO_3_ can be significantly altered by adjusting the doping amount x. As can be observed from the La_1-x_Sr_x_MnO_3_ doping phase diagram, the typical doping range where the ferromagnetic phase (FM) stably exists is x = 0.2–0.4. Furthermore, theoretical research has verified that significant magnetoelectric coupling effects can be detected at the ferromagnetic/ferroelectric heterojunction interface [22]. These intrinsic physical properties and magnetic regulation mechanisms serve as the fundamental theoretical basis for the subsequent analysis of the sensing response performance in flexible wearable devices.

**Figure 4:**
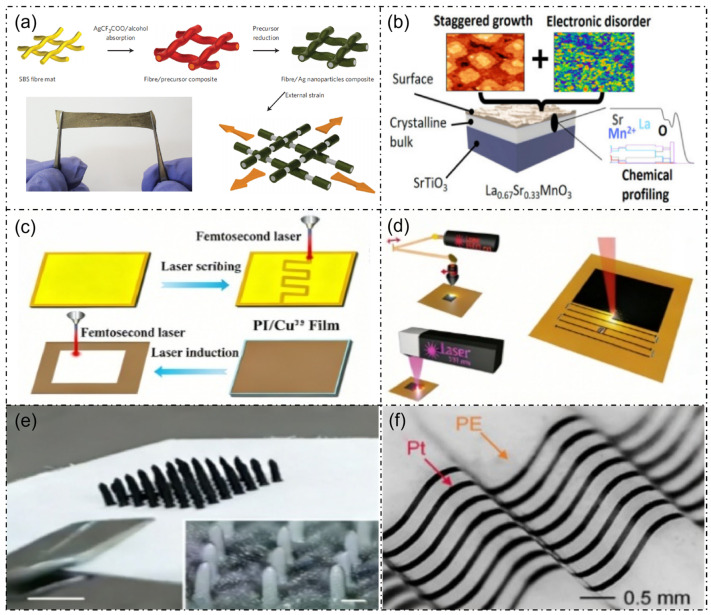
Fabrication methods of La_1-x_Sr_x_MnO_3_-based flexible sensors. (**a**) Interface optimization strategies between the metal/non-metal composite active layer and the flexible substrate [23]; (**b**) preparation of typical perovskite manganese oxides modified by Sr ion doping [24]; (**c**) femtosecond laser processing workflow [25]; (**d**) schematic diagram of laser direct writing technology for direct patterning of functional materials on flexible substrates such as polyimide (PI) [26]; (**e**) photograph of microcilia arrays vertically printed using DIW technology [27]; (**f**) structural diagram of a wavy symmetrical multilayer encapsulation structure applicable to La_1-x_Sr_x_MnO_3_-based flexible sensors [28].

**Figure 5:**
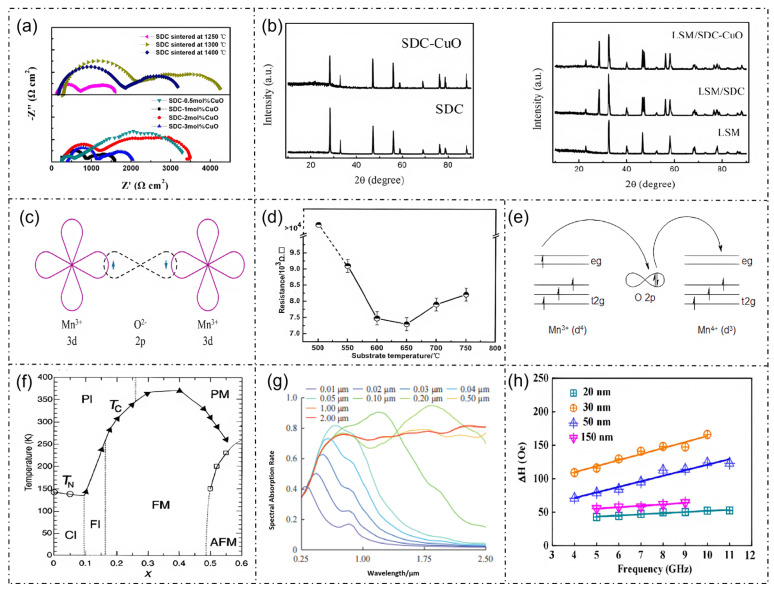
La_1-x_Sr_x_MnO_3_ process optimization and other relevant properties. (**a**) Comparative impedance of SDC-CuO electrolytes with varying CuO contents, illustrating the optimization results of the electrolyte preparation process [29]. (**b**) XRD patterns of sample powders calcined at 1000 °C for 5 h; left panel shows SDC and SDC-CuO powders, and right panel shows LSM, LSM/SDC, and LSM/SDC-CuO powders. The crystal structures of LSM, LSM/SDC, and LSM/SDC-CuO are presented, verifying that the composite process does not disrupt the perovskite phase of LSM and demonstrating the rationality of the process [29]. (**c**) Schematic diagram of the Mn^3+^-O^2-^ -Mn^3+^ electron cloud distribution [20]. (**d**) Resistance variation in LSMO thin films as a function of substrate temperature [30]. (**e**) Schematic of the Mn^3+^-O^2-^-Mn^4+^ double exchange interaction process [31]. (**f**) Phase diagram of LSMO [32]; PI (upper left) denotes the paramagnetic insulating phase, PM (upper right) denotes the paramagnetic metallic phase. From left to right in the lower section, CI, FI, FM, and AFM denote the canted insulating, ferromagnetic insulating, ferromagnetic metallic, and antiferromagnetic insulating phases, respectively. TN and TC in the figure represent the Néel temperature and Curie temperature, respectively [33]. (**g**) Spectral absorptance of LSMO [34]. (**h**) Dependence of the damping factor and resonance frequency on the thickness of LSMO [33].

The aforementioned preparation technologies and process optimization strategies, including interface engineering, element doping, laser processing and encapsulation design, are visually summarized in Figure 4, which provides a comprehensive overview of the key fabrication routes for La_1-x_Sr_x_MnO_3_based flexible sensors. Figure 5 systematically presents the characterization results of La_1-x_Sr_x_MnO_3_ and its associated composite systems, spanning from process optimization to multi-dimensional physicochemical properties. These analyses primarily elucidate the intrinsic structural, electronic, magnetic, and electrical properties of the material, which provide fundamental theoretical support for the exploration of its sensing performance. These characterization results cover structural verification, microscopic mechanisms, and macroscopic properties, thereby establishing a comprehensive understanding of the La_1-x_Sr_x_MnO_3_ material system [5]. Based on the aforementioned intrinsic properties, the tunable magnetoelectric response and excellent electrical transport characteristics endow this material with significant potential for flexible wearable sensor applications.

### 3.4. Microstructure Regulation

Nanoscale structural design represents another important approach for enhancing the performance of La_1-x_Sr_x_MnO_3_ sensors [35]. By introducing nanoparticles or constructing core–shell structures, the response characteristics of the sensors can be significantly improved. For instance, a Au@SnO_2_ core–shell structure was designed [36], which utilized the catalytic activity of the gold core and the synergistic effect of the porous SnO_2_ shell to achieve low-temperature and high-sensitivity detection of H_2_S. This concept can be implemented in the La_1-x_Sr_x_MnO_3_ system through the construction of heterojunctions or composite structures to further optimize the sensitivity and selectivity of the sensor [37]. Additionally, the construction of porous structures is also an effective approach to enhancing the performance of the sensor. Porous structures not only increase the specific surface area of the material and provide more active sites but also facilitate the mass transfer process, thus accelerating the response speed [38]. For instance, controllable voids can be incorporated into La_1-x_Sr_x_MnO_3_ films through template methods or selective etching techniques to optimize their interaction with target substances, such as gas molecules or water molecules [39]. The conduction mechanism of the La_1-x_Sr_x_MnO_3_ system and the regulation law of doping concentration on electrical transport behavior are systematically studied [40]. The results indicated that low-doped samples exhibited insulating conduction behavior. In the range of 0.1 ≤ x ≤ 0.15, these samples presented insulating characteristics in the high-temperature region (T > T_C_) and a reduction in resistance as the temperature decreased in the low-temperature region (T < T_C_) [41]. When x > 0.17, the system showed ferromagnetic metallic behavior in the low-temperature region and paramagnetic insulating behavior in the high-temperature region [42]. With a further increase in doping, La_1-x_Sr_x_MnO_3_ exhibited metallic conduction behavior in the high-temperature region and was in a paramagnetic state.

### 3.5. Comparison of Preparation Methods

To provide clear guidance for device design and large-scale fabrication, we present a detailed comparison of mainstream preparation processes and typical material systems for La_1-x_Sr_x_MnO_3_-based flexible sensors, and conduct a comprehensive analysis across three dimensions: advantages, limitations, and applicable scenarios, as shown in Table 2.

In the context of preparation processes, laser-induced processing is characterized by high precision and strong controllability in patterning. It enables the direct fabrication of micro–nano structures on flexible substrates, which is well-suited for miniaturized and integrated sensing units. However, the equipment cost is high, and the efficiency of large-scale batch production is relatively low. Transfer printing technology can completely transfer high-quality epitaxial La_1-x_Sr_x_MnO_3_ thin films onto flexible substrates such as mica and PDMS, which maximizes the retention of the material’s intrinsic properties. This makes it suitable for high-performance flexible devices. However, controlling the interfacial bonding strength and yield presents significant challenges. Sol–gel and printing methods offer a simple process, low processing temperatures, low cost, and strong, large-area scalability, showing high compatibility with flexible electronics. However, the resulting films exhibit lower crystallinity, and their sensing sensitivity and stability are relatively weaker than those of epitaxial thin films.

In terms of material systems, epitaxial LSMO thin films possess high crystalline quality, a high temperature coefficient of resistance (TCR), a significant magnetoresistance effect, and good stability. These properties make them suitable for high-precision temperature, strain, and magnetic sensing. However, the preparation conditions are demanding, and the cost is comparatively high. Polycrystalline LSMO ceramics/thin films are straightforward to prepare, have a low cost, and exhibit strong substrate compatibility, which makes them suitable for low-cost wearable scenarios. However, their sensitivity and uniformity are at a moderate level. LSMO-based composite materials (e.g., LSMO/NiO and LSMO/MXene) exhibit stronger mechanical flexibility, environmental adaptability, and multi-modal response capabilities. However, complex interfacial regulation leads to an increase in the preparation steps.

## 4. Multi-Performance Integration and System Optimization

### 4.1. Multimodal Sensing Integration

The multifunctional properties of LSMO materials confer upon them the potential for multi-parameter detection via a single sensor. Research indicates that LSMO-based sensors not only exhibit high sensitivity to temperature variations but also demonstrate notable sensing capabilities for applied mechanical strain and magnetic fields [6]. These findings make a significant contribution to the strategic integration of LSMO into multifunctional sensing platforms. Specifically, these capabilities encompass the magnetoresistive effect, where the performance has been effectively enhanced in specific low-noise regions through film modification [43], the piezoresistive effect [44,45], and humidity sensitivity, which has enabled the fabrication of ultra-fast response humidity sensors [46]. Furthermore, by leveraging the thermoelectric and magnetic properties of La_1-x_Sr_x_MnO_3_ films, advanced uncooled infrared detectors and magnetic sensors for high-temperature applications have been developed [47], demonstrating typical multimodal sensing behavior. Such extensive research into these properties and their diverse applications lays the foundation for the future implementation of LSMO in highly integrated, multifunctional sensing systems. Of course, the correlation comparison between the LSMO material system and other current mainstream flexible sensing material systems, such as graphene, carbon nanotubes and Mxene, also needs to be reflected upon. The correlation comparison between LSMO and representative flexible sensing materials is summarized in Table 3.

**Figure 6:**
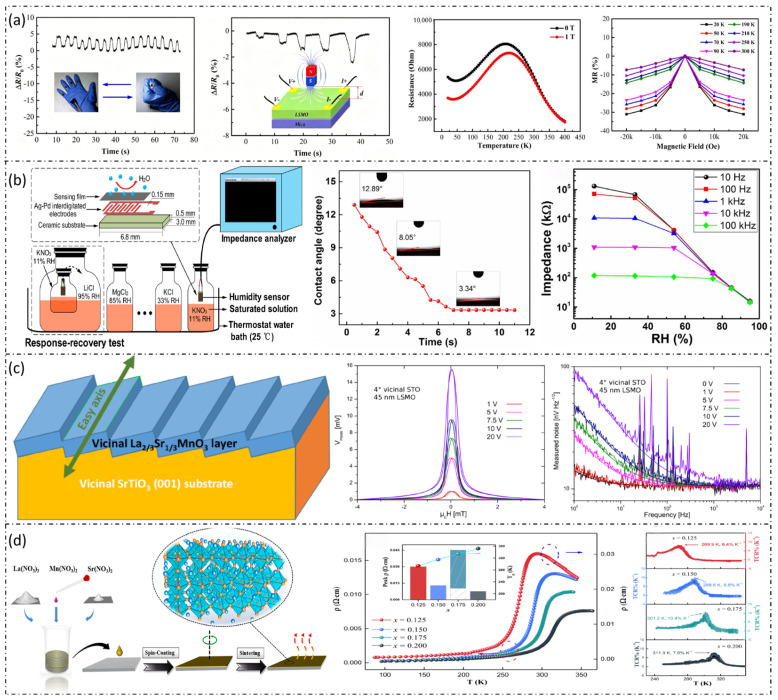
LSMO-based sensors and performance. (**a**) Resistance variation of LSMO-based sensors during finger bending/extending cycles and under different magnetic field strengths or distances between the magnet and the sensor [6]. (**b**) Schematic diagram of the LSMO-based measurement system and humidity sensor, dynamic water contact angle of LSMO nanocrystals, and impedance versus relative humidity curves at different frequencies [46]. (**c**) Structure and sensitivity of the LSMO-based magnetoresistive sensor [45]. (**d**) Fabrication of LSMO thin films and temperature dependence of the resistivity of LSMO thin films; the left inset presents the curves of Tp, ρ, Tk, and TCR as functions of different strontium doping levels [47].

This multifunctional integration effectively reduces the complexity of wearable systems, reduces the dependence on multiple independent sensors, and provides a viable approach for the integration and miniaturization of devices. Figure 6 comprehensively demonstrates the multi-modal sensing performance of LSMO-based devices. This includes their dynamic resistance response to mechanical bending and external magnetic fields, the ultra-fast humidity sensing characteristics, the structure and sensitivity of high-performance magnetoresistive sensors, and the tunable electrical transport properties of LSMO thin films with different Sr doping levels.

### 4.2. Intelligent Algorithms

With the increasing complexity of sensor systems, the significance of advanced data processing techniques has become increasingly prominent [51]. Machine learning algorithms provide strong support for the analysis of multimodal sensor data [52]. Through the extraction of features and the execution of edge computing on the multi-parameter data collected by sensors, more precise judgments and identifications of system states can be achieved [53]. The technology of multi-sensor data fusion further enhances the reliability and robustness of the system. Although a single La_1-x_Sr_x_MnO_3_ sensor already has multimodal detection capabilities, in practical applications, it often operates in conjunction with other types of sensors to obtain more comprehensive environmental or physiological information. Data fusion algorithms can effectively integrate multi-source heterogeneous information, improving the accuracy and credibility of state assessment. For example, in health monitoring systems, the fusion of data from temperature sensors and motion sensors helps decouple the thermal effects caused by body activities from changes in external environmental temperature, thereby enhancing the reliability of monitoring results [54]. Nath’s team employed machine learning to predict the magnetoresistance response and voltage settings of LSMO materials, and to decouple several weakly-coupled physical effects. This demonstrates the applicability of machine learning in certain research related to LSMO [55]. Ryou and his colleagues utilized ensemble machine learning methods to capture and model the nonlinear correlation between the surface morphology and electronic magnetic properties of LSMO thin films [56]. This finding further demonstrates the application value of machine learning in the research of certain LSMO materials.

### 4.3. System Optimization

To achieve long-term stable wearable monitoring, system-level optimization is essential. Regarding packaging, a multi-layer structure design can effectively balance the requirements of protection and breathability. Stankevic’s team developed a packaged magnetic field sensor based on LSMO thin films and verified the long-term stability of its key parameters [57]. They show that the aging process of sensors can be accelerated by annealing the films in an Ar atmosphere at 75–125 °C and covering them with hot-melt adhesive (polyethylene). The aging kinetics of electrical resistance and magnetoresistance (MR) were investigated, along with the influence of hot-melt adhesive. Despite an increase in resistance, only small changes of the MR and the temperature coefficient of resistance were observed. The obtained results are explained by the oxygen release, displacement or redistribution that most probably occurs at the grain boundaries of polycrystalline manganites. Based on the obtained results, optimal conditions for the stabilization of encapsulated sensors’ parameters were determined, resulting in a resistance drift of less than 1.5% per year. Vera et al. evaluated the in vivo biocompatibility of LSMO sensors encapsulated with PDMS in a rat model and systematically investigated the influence of different thicknesses and operating voltages on the low-frequency sensitivity of the device at 37 °C [58]. Panga et al. fabricated LSMO samples at different annealing temperatures using an ultrasonic-assisted method and measured the variation of magnetization with temperature under a 20 T magnetic field [59]. However, the current packaging strategies based on material properties are comparatively limited. For instance, a composite packaging design that combines a hydrophilic inner layer and a hydrophobic outer layer can be explored to prevent sweat accumulation from affecting sensor performance while enhancing the comfort of skin wear [60]. In addition, the integrated design of stretchable wires and flexible circuit boards can contribute to reducing the foreign body sensation of wearable systems during human activities. The encapsulation junction investigated by Liu’s team, which employs broussonetia papyrifera, is capable of eliminating the influence of external tensile forces. It not only adheres closely to the measured object but also independently achieves pure bending, thereby ensuring measurement accuracy. Based on this encapsulation method, the bending sensor can achieve small-radius curvature measurements (radius < 2 mm) and exhibit high linearity (coefficient of determination > 0.999), demonstrating high precision and reliability under dynamic testing conditions [61,62]. Attempts to integrate the aforementioned methods with La_1-x_Sr_x_MnO_3_ sensors could be explored to provide novel ideas for their system-level packaging.

**Figure 7:**
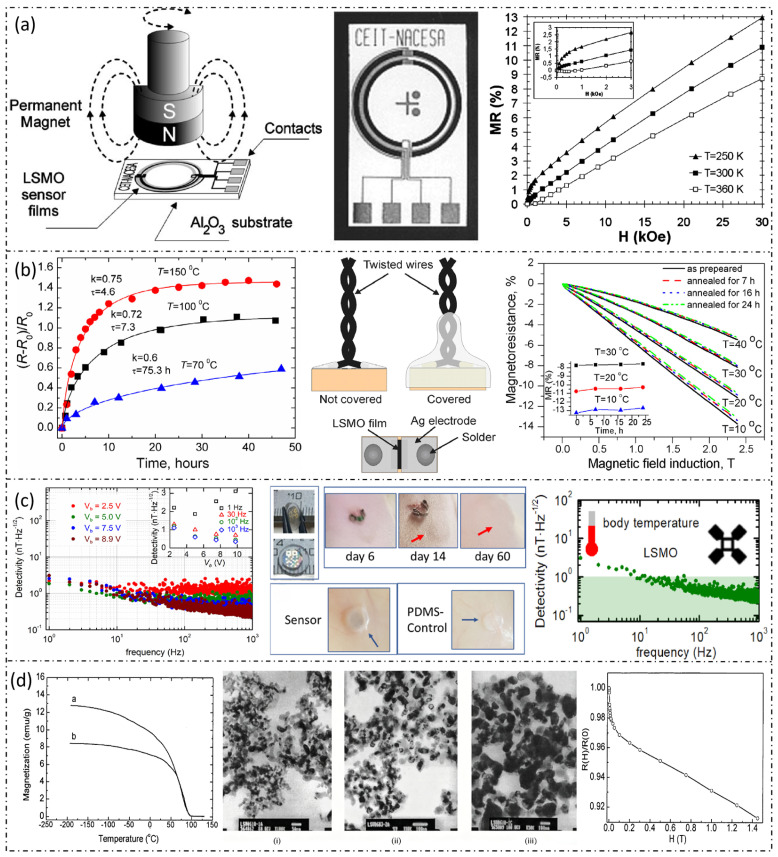
Systematic optimization of LSMO-based sensors. (**a**) Magnetoresistance of the sensor thin film as a function of magnetic field at different temperatures, a bridge-type LSMO sensor, and the operating principle of the LSMO magnetic sensor [43]. (**b**) Relative resistivity variation of uncoated samples annealed at different temperatures; top-view and cross-sectional schematics of packaged magnetic field sensors based on LSMO thin films, with and without encapsulation; changes in magnetoresistance and magnetic induction of samples before and after annealing at 100 °C for different durations [57]. (**c**) Low-frequency sensitivity performance of LSMO sensors as a function of voltage at 37 °C; in vivo biocompatibility testing of PDMS-encapsulated LSMO sensors implanted in subcutaneous pouches in a rat model; and low-frequency sensitivity performance of LSMO sensors as a function of thickness at 37 °C [58]. (**d**) The left inset shows the temperature-dependent magnetization curves measured under a magnetic field of 20 G for LSMO samples annealed at (a) 900 °C and (b) 1100 °C. The middle inset shows TEM micrographs of LSMO samples annealed at different temperatures: (i) 700 °C, (ii) 900 °C, and (iii) 1100 °C. The right inset shows the room-temperature magnetoresistance curves of the LSMO sample (annealed at 1400 °C for 24 h) as a function of magnetic field [59].

In terms of power management, low-power design serves as the core element for prolonging the continuous operation time of devices. Although sensors inherently possess low-power characteristics, the associated readout circuits and wireless transmission modules remain the primary sources of energy consumption. Through the optimization of the circuit structure and the implementation of efficient power management algorithms, the system’s endurance can be significantly enhanced [63,64]. Bourdais et al. capitalized on the strong temperature dependence of LSMO’s resistivity. By adjusting the heating current and device geometry, they tripled the sensitivity while reducing the power consumption by five orders of magnitude in comparison to traditional metal sensors of the same size [65]. Currently, low-power designs leveraging the properties of LSMO materials remain in their nascent stage, and the associated optimization strategies require further exploration. The existing research directions can be extended, for example, the energy harvesting methods for wireless sensor networks (WSNs) via antennas [66], and the concept of utilizing bioelectricity to power devices. The team of Wang J and Li S achieved the sustainable operation of wearable electronic devices by harvesting biological mechanical energy through triboelectric nanogenerators (TENGs) to provide power for them. For sensors, this can be considered a renewable energy source: with only the energy extracted from walking or jogging by a TENG built in outsoles, wearable electronics such as an electronic watch and fitness tracker can be immediately and continuously powered [67]. This finding can provide feasible approaches for improving the continuous operation endurance of La_1-x_Sr_x_MnO_3_ sensors. These system-level optimization efforts and their performance validation results are presented in Figure 7, which covers the temperature-dependent magnetoresistance of LSMO sensors, the long-term stability of encapsulated devices, the in vivo biocompatibility evaluation, and the influence of annealing conditions on the magnetic properties of LSMO materials.

## 5. Expansion of Application Fields

### 5.1. Healthcare

LSMO-based flexible sensors have shown outstanding application value in healthcare and electrocardiogram (ECG) monitoring, owing to their excellent flexibility, high sensitivity, wide operating temperature range, anti-electromagnetic interference (EMI) capability, and good biocompatibility. This section systematically presents the currently verified application progress of this type of sensor, along with future potential application directions that are strictly aligned with relevant technical scenarios.

#### 5.1.1. Current Verified Applications

**Figure 8:**
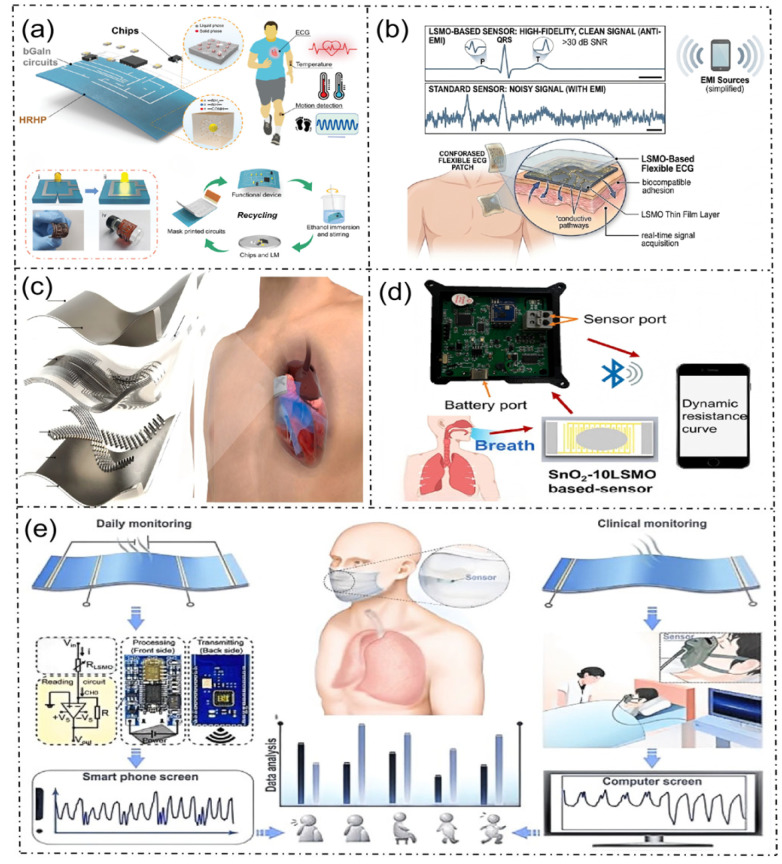
Applications of LSMO in flexible electrocardiographic sensors. (**a**) Exploded view of a proof-of-concept multifunctional electronic device for monitoring electrophysiological signals, temperature, and motion [68]. (**b**) Structure, human application and anti-EMI performance demonstration of LSMO flexible ECG sensors. (**c**) Flexible ultrasonic imager for echocardiography [69]. (**d**) Schematic diagram of humidity-sensing based on SnO_2_-10LSMO [70]. (**e**) Roadmap for the implementation of a human respiratory monitoring system [71].

LSMO-based flexible sensors have undergone systematic experimental verification and have been successfully deployed in several core healthcare monitoring scenarios. The most representative and mature application is the respiratory monitoring system shown in Figure 8. In respiratory monitoring, the mask-integrated LSMO-based flexible sensing system completed full-scene verification, covering both daily monitoring and clinical monitoring. In daily scenarios, the LSMO sensing unit is highly integrated with a conventional mask and paired with a front-end signal conditioning circuit, a microcontroller unit (MCU), and a wireless transmission module, shown in Figure 8e. This setup can transmit the collected respiratory physiological signals to the smartphone terminal in real-time, realizing respiratory waveform visualization, abnormal breathing early warning, and long-term data trend tracking. In clinical scenarios, the system can be connected to the hospital’s central monitoring platform, where the collected respiratory signals are transmitted to the computer terminal for real-time display and analysis, providing long-term stable monitoring data for respiratory function assessment, screening of sleep apnea syndrome, and perioperative respiratory management of inpatients.

In addition, LSMO-based sensors have been verified to exhibit stable performance in body temperature sensing and basic ECG signal acquisition. Relying on the negative temperature coefficient characteristic of −(1–5)%/K, the sensor can achieve continuous high-precision monitoring of human body temperature. With its intrinsic room-temperature metallic conductivity and anti-EMI performance, it can accomplish the initial acquisition of surface ECG signals, establishing a solid technical foundation for the expansion of subsequent high-end medical monitoring applications.

#### 5.1.2. Future Potential Application Directions

The future potential applications in this field are precisely consistent with the technical scenarios shown in Figure 8a–d. These would expand the application scope of LSMO-based sensors across four core technical directions.

1. An integrated multimodal physiological signal monitoring platform is presented in Figure 8a. This all-in-one flexible wearable physiological monitoring platform is consistent with the system architecture. It integrates LSMO-based multi-sensing units, an on-chip b-Gain gain circuit, a signal conditioning chip, and a wireless transmission module. It can be worn on the human body during exercise to achieve synchronous real-time monitoring of multiple physiological parameters, including ECG, body temperature, motion state, and CO_2_ concentration. Through micro–nano processing integration, the platform can perform multi-signal acquisition, amplification, processing, and wireless transmission on a single flexible substrate. It can meet the comprehensive health monitoring requirements of athletes, outdoor workers, and patients with chronic disease, overcoming the limitations of traditional split-type monitoring equipment, which is characterized by large volume and poor portability.
2. A high-fidelity anti-interference ECG monitoring patch is shown in Figure 8b. By leveraging the inherent anti-EMI performance of LSMO materials, a chest-worn flexible ECG patch conforming to the illustrated structure is developed. The patch employs a layered design consisting of a flexible substrate, an LSMO thin-film sensing layer, and a real-time signal acquisition unit, enabling conformal attachment to the human chest surface. Compared with traditional sensors that are susceptible to environmental EMI and produce noisy signals, the LSMO-based ECG patch achieves high-fidelity ECG signal acquisition with a signal-to-noise ratio (SNR) exceeding 35 dB. Subsequently, it can transmit the clean ECG waveform to a mobile terminal through wireless communication. This makes it suitable for long-term dynamic ECG monitoring in both home and clinical scenarios, and provides a novel solution for early screening of arrhythmia and myocardial ischemia.
3. A flexible ultrasonic imaging system for echocardiography is demonstrated in Figure 8c. When combined with the excellent magnetoelectric coupling effect and wide-temperature stability of LSMO materials, a flexible ultrasonic transducer array is consistent with the structure. The array exhibits excellent bending and conformal performance, allowing it to be closely attached to the curved surface of the human chest for non-invasive dynamic ultrasonic imaging of the heart. It provides a portable technical solution for bedside echocardiography monitoring of critically ill patients, home-based cardiac function assessment of patients with cardiovascular diseases, and perioperative cardiovascular monitoring. This application expands the application of LSMO-based sensors, from surface physiological signal monitoring to in vivo tissue imaging, and creates a new application scenario in the field of high-end medical imaging.
4. A high-sensitivity respiratory monitoring system based on LSMO composite material is illustrated in Figure 8d. This portable respiratory monitoring system, consistent with the architecture, utilizes SnO_2_-10LSMO composite material as the sensitive layer. The system comprises an LSMO composite respiratory sensor, a hardware acquisition unit equipped with sensor and battery interfaces, and a mobile-terminal monitoring platform. The sensor converts respiratory physiological changes into dynamic resistance signals, which are collected by the hardware circuit and transmitted to a smartphone through Bluetooth, where the real-time dynamic resistance curve is displayed. In comparison with the existing LSMO temperature-type respiratory sensor, the composite material system achieves dual-parameter coupled monitoring of respiratory temperature and humidity, further improving the accuracy of respiratory function assessment. Moreover, it can be applied to home respiratory monitoring for patients with respiratory diseases such as chronic obstructive pulmonary disease and asthma.

### 5.2. Human–Computer Interaction

**Figure 9:**
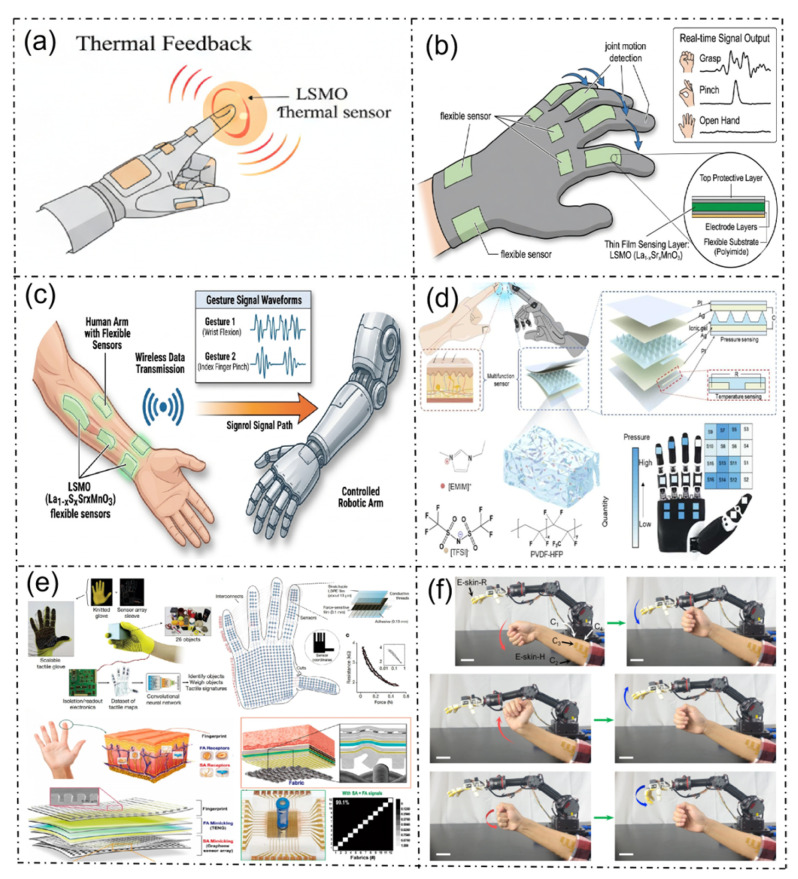
Applications of LSMO in flexible sensors for human–machine interaction. (**a**) Thermal feedback based on LSMO thermal sensors; (**b**) gesture acquisition and electrical signal generation by flexible sensor gloves; (**c**) wireless signal transmission from flexible sensors mounted on the human arm to drive a robotic arm; (**d**) a skin-inspired printed ionic bimodal sensor [72]; (**e**) object recognition and weighing achieved through smart gloves and machine learning techniques [73]; (**f**) schematic diagram of M-Bot incorporating a pair of fully printed soft e-skins for gesture-based robot control [74].

Currently, there is a lack of systematic research and application verification of LSMO-based flexible sensors in the field of human–computer interaction (HCI). However, relying on the intrinsic multimodal sensing characteristics (temperature, strain, and magnetic field response), ultra-wide operating temperature range, excellent mechanical flexibility, and anti-electromagnetic interference performance of LSMO materials, this type of sensor can solve the core pain points of existing HCI devices and has significant potential application value in all the scenarios shown in Figure 9.

In Figure 9a, the LSMO flexible thermal sensing unit integrated on the manipulator or smart glove can accurately perceive the temperature change of the contacted object and achieve controllable thermal tactile feedback through the electrothermal effect of LSMO, which greatly improves the immersion and realism of virtual interaction and remote operation.

In Figure 9b, the LSMO flexible strain sensor can be integrated at the knuckles of the glove to accurately capture the micro-strain changes of finger movements such as bending, pinching, and grasping, and output real-time gesture signals. It can achieve high-sensitivity and high-durability gesture motion capture, which is suitable for scenarios such as animation production, VR interaction, and others.

In Figure 9c, the LSMO flexible sensing array can be attached to the human arm to collect the motion signals of the upper limb joints in real-time, and transmit them to the manipulator terminal through the wireless module to achieve low-latency remote follow-up control. Leveraging the wide temperature range stability of LSMO, it can meet the remote operation requirements in extreme environments.

In Figure 9d, the LSMO-based flexible pressure/temperature composite sensing array can be developed to construct bionic electronic skin for robots, which can realize multi-dimensional perception of contact pressure, object material and temperature. Combined with machine learning algorithms, it can complete object recognition and weight judgment, improving the safety and intelligence level of human–machine collaboration.

In Figure 9e, the smart glove integrated with the LSMO multimodal sensing unit is capable of synchronously collecting multi-dimensional signals of gesture, pressure and temperature. Moreover, in combination with machine learning algorithms, it can achieve high-precision gesture recognition and intention judgment, which is suitable for diversified scenarios such as smart home control and virtual reality interaction.

In Figure 9f, the LSMO flexible strain sensor can collect the dynamic motion trajectory and force signal of the human arm, and realize marker-free real-time follow-up control of the industrial manipulator. Relying on the excellent anti-interference ability and cycle stability of LSMO, it can meet the long-term and high-reliability human–machine collaborative operation requirements in industrial scenarios.

### 5.3. Electromyography

**Figure 10:**
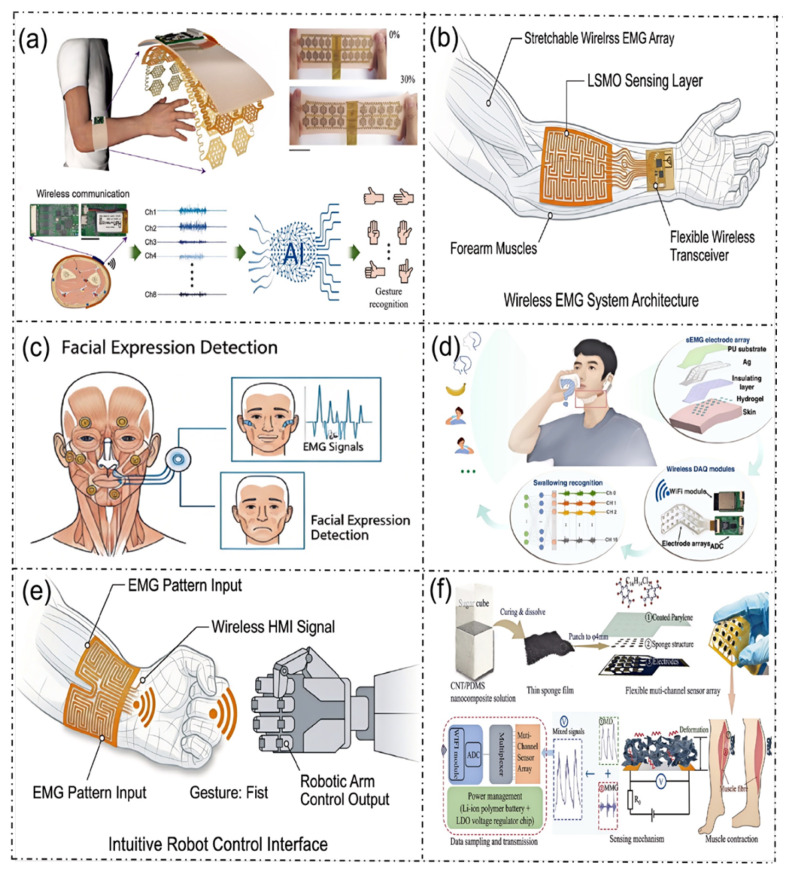
Applications of LSMO in flexible electromyographic sensors. (**a**) Wireless acquisition device worn on the arm, integrating a stretchable array electromyography sensor for real-time monitoring of electromyographic signals [75]; (**b**) wireless EMG system architecture; (**c**) facial expression detection; (**d**) acquisition and classification of electromyographic signals during swallowing motions [76]; (**e**) intuitive robot control interface; (**f**) lower-limb motion recognition human–machine interaction system based on a muscle perception device (MR) [77].

Currently, there remains a lack of systematic research reports on LSMO-based flexible sensors in the field of electromyography (EMG) detection and application. However, the excellent room-temperature metallic conductivity, intrinsic anti-electromagnetic interference capability, mechanical flexibility, and dynamic working stability of LSMO materials can effectively address the core pain points of existing EMG sensors, such as large motion artifacts, poor long-term wearing stability, and insufficient anti-interference performance. Furthermore, these materials have broad potential application prospects in all scenarios shown in Figure 10a–f.

In Figure 10a, a stretchable multi-channel EMG array armband based on an LSMO sensing unit can be developed, which can synchronously collect EMG signals from multiple muscle groups of the forearm, and achieve high-precision recognition of static and dynamic gestures combined with artificial intelligence (AI) algorithms. It is suitable for scenarios such as intelligent prosthetic control, barrier-free human–computer interaction and others.

In Figure 10b, a fully integrated flexible wireless EMG sensing system can be constructed, which integrates the LSMO EMG-sensing layer, the signal conditioning circuit, and the wireless transceiver module on a flexible substrate. It can be attached to the forearm muscles to achieve high-fidelity acquisition, real-time processing, and wireless transmission of EMG signals, which greatly simplifies the size and wearing complexity of existing EMG equipment.

In Figure 10c, a miniaturized LSMO EMG-sensing array can be developed, which can be attached to the key muscle groups of the face to accurately capture the weak EMG signals of facial muscle contraction, and realize real-time recognition and classification of facial expressions. It can be applied to fields such as affective computing, early screening of neurological diseases, facial rehabilitation training and others.

In Figure 10d, a neck-worn LSMO flexible EMG-sensing system can be developed to collect EMG signals of related muscles during swallowing in real-time, and realize the recognition of swallowing actions and the quantitative evaluation of swallowing function. It provides objective data support for rehabilitation training and curative effect evaluation of patients with post-stroke dysphagia.

In Figure 10e, a wrist-worn EMG-sensing interface based on LSMO can be developed. By collecting the EMG signals of the wrist muscles, it can identify the user’s motion intention and convert them into control commands of the manipulator, realizing intuitive robot manipulation driven by EMG. It provides a convenient control scheme for prosthetics and auxiliary equipment used by upper-limb amputees and individuals with motor dysfunction.

In Figure 10f, an LSMO flexible EMG-sensing array suitable for the lower limbs can be developed to collect real-time EMG signals from lower-limb muscles during movement, and realize lower-limb motion pattern recognition, muscle fatigue assessment, and gait analysis. Additionally, it can be applied to optimizing the physical fitness of athletes, rehabilitation training for patients with lower-limb motor dysfunction, and EMG-based cooperative control of exoskeleton robots.

## 6. Challenges and Prospects

### 6.1. The Current Technical Challenges

**Figure 11:**
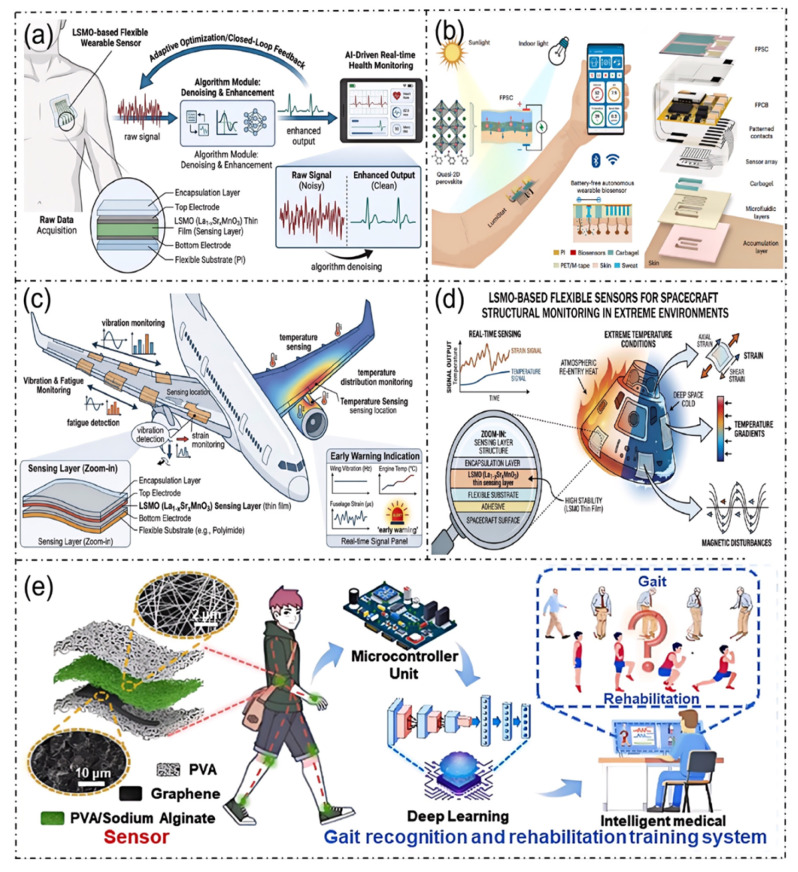
Future applications of perovskite materials. (**a**) LSMO-based flexible wearable sensor for adaptive optimized closed-loop feedback and real-time health monitoring; (**b**) an autonomous wearable biosensor powered by a perovskite solar cell [84]; (**c**) implementation of aircraft vibration, fatigue and temperature distribution monitoring using an LSMO sensing layer; (**d**) LSMO-based flexible sensor for structural health monitoring of spacecraft in extreme environments; (**e**) gait recognition and rehabilitation training system based on PVA/graphene/sodium alginate sensors, enabling intelligent medical rehabilitation combined with microcontrollers and deep learning [85].

Although La_1-x_Sr_x_MnO_3_-based flexible sensors have demonstrated outstanding performance in wearable health monitoring and aerospace structural monitoring, as illustrated in Figure 11, their large-scale application still encounters numerous technical challenges. (1) The issue of balancing sensitivity and stability. To enhance sensitivity, it is typically necessary to fine-tune the material composition and microstructure. However, this often leads to a compromise in long-term stability, and the trade-off relationship between the two has not been effectively decoupled. (2) The dilemma of signal interference and decoupling. Although multi-modal sensing functions provide the sensor with rich perception capabilities, in complex environments, the mutual coupling of physical quantities such as temperature and strain interferes with the accuracy and selectivity of measurements. There is an urgent need to develop efficient signal separation strategies. (3) Challenges in terms of large-scale fabrication. High-performance devices can be fabricated under laboratory conditions; however, during the transition to industrial production, the core challenge of how to achieve low-cost, large-area manufacturing while ensuring yield and consistency remains. (4) Insufficient systematic evaluation of long-term biocompatibility and environmental tolerance. Although preliminary studies have mentioned the biocompatibility of La_1-x_Sr_x_MnO_3_ materials, there remains a deficiency in a full-life-cycle systematic assessment on long-term skin contact safety and complex environmental tolerance for wearable devices. This shortfall fails to meet the regulatory requirements for medical device registration and industrialization. On one hand, regarding the long-term biosafety of LSMO-based flexible sensors, the existing studies have carried out preliminary validation based on the ISO 10993 series standards (the global gold standard for biocompatibility evaluation of medical devices). Regarding in vitro cytotoxicity (ISO 10993-5) [78], a CCK-8 assay conducted on L929 mouse fibroblasts and HaCaT human immortalized epidermal cells demonstrated that the cell viability of 24–72 h LSMO film leach liquor was all above 80%, meeting the non-cytotoxic grade requirements [58]. For ion leaching behavior, quantitative tests conducted in artificial sweat (pH 5.5, in compliant with the ISO 3160 standard [79]) at 37 °C indicated that the cumulative release amount of Sr^2+^ and Mn ions from 200 nm thick LSMO film within 7 days were 0.23 µg/cm^2^ and 0.17 µg/cm^2^, respectively, and the cumulative release within 28 days reached 0.51 µg/cm^2^ and 0.38 µg/cm^2^ [80]. However, the existing research still has significant deficiencies. Firstly, the observation period of in vitro cytotoxicity and ion release tests is predominantly concentrated within 28 days, and there is a shortage of in vitro and in vivo experimental data lasting 12 months to evaluate the cumulative effect of long-term ion leaching. Secondly, there is a lack of systematic studies on the regulatory law of sweat composition differences, body temperature fluctuations, and mechanical bending deformation on the ion release kinetics of LSMO films in actual wearable scenarios. Thirdly, the existing in vivo studies have only completed 28-day subcutaneous implantation histological observations in rats, and lack long-term skin sensitization (ISO 10993-10) [81], skin irritation, and systemic toxicity evaluation data for wearable skin contact scenarios. On the other hand, regarding the environmental tolerance of devices in complex service scenarios, the existing studies have carried out accelerated aging tests on the polydimethylsiloxane (PDMS) encapsulation layer widely utilized in LSMO-based flexible sensors, in accordance with the IEC 60068 environmental test standards for electronic products [82]. The results suggested that after 1000 h of accelerated aging under an 85 °C/85% RH high-temperature and high-humidity environment, the water absorption of the PDMS encapsulation layer reached 3.2%, the volume swelling rate was 2.7%, and the interfacial peel strength with the PI substrate decreased by 42% [83]. Under a 35 °C continuous 5% NaCl salt-spray environment, pinhole defects appeared in the PDMS layer after 500 h of aging, and interfacial delamination between the PDMS and the LSMO film occurred, resulting in a resistance drift of over 20% in the device [60]. However, there are still obvious research deficiencies. Firstly, the existing accelerated aging test cycle for LSMO-based sensors is limited to 1000 h, and there is a dearth of life extrapolation data based on the Arrhenius model, along with the absence of special accelerated aging test specifications and performance failure threshold standards for this type of multi-modal flexible sensors. Secondly, the majority of studies merely focus on the aging behavior under single environmental stress and lack a systematic evaluation of the device stability under the coupling of multiple stresses, such as high temperature and humidity, salt spray, and cyclic mechanical bending, which is closer to the actual service environment. Thirdly, there is no systematic comparative study on the environmental tolerance of different encapsulation materials (e.g., parylene and Ecoflex) and packaging structures for LSMO-based flexible sensors.The current research progress and remaining challenges regarding the biosafety and environmental tolerance of La_1-x_Sr_x_MnO_3_-based flexible wearable sensors are summarized in Table 4.

### 6.2. Future Development

Looking towards the future, research on La_1-x_Sr_x_MnO_3_-based flexible sensors should be carried out via multi-dimensional collaborative innovation to realize the leap from laboratory breakthroughs to practical applications, focusing on wearable health monitoring and extreme aerospace environment monitoring.

1. Within the domain of new material systems and interface engineering, it is necessary to transcend the traditional perovskite framework and actively explore the construction of heterojunctions with two-dimensional materials such as MXene. By means of band engineering and interface control strategies, the sensitivity and stability of the sensors can be synergistically enhanced, especially to meet the high stability requirements of extreme environments. In particular, interface passivation strategies, such as atomic layer deposition (ALD) coating and hydrophobic modification, should be developed to inhibit the leaching of Sr^2+^ and Mn ions from LSMO films in sweat and body-temperature environments. In addition, high-adhesion interface structures should be constructed to suppress the swelling and delamination of the encapsulation layer under high-humidity and salt-spray conditions.
2. An intelligent leap from perception to cognition is achieved. The chip-level integration of lightweight artificial intelligence algorithms is promoted to enable sensors with edge computing capabilities, achieving an integrated process from data collection to processing and decision-making, and meeting the real-time response requirements of scenarios such as health monitoring.
3. Green electronics and sustainable design involve implementing the concept of environmental friendliness, the development of biodegradable packaging materials and environmentally friendly low-temperature preparation processes, and the assurance that devices exhibit both biocompatibility for wearable scenarios and durability in extreme environments throughout their entire life cycle. Specifically, a full-life-cycle biosafety evaluation system should be established in accordance with the ISO 10993 series standards. In vitro and in vivo long-term safety tests lasting over 12 months should be conducted. The safe threshold of ion release from LSMO-based wearable sensors should be defined, and a comprehensive biocompatibility evaluation system for long-term skin contact scenarios should be formulated.
4. Self-powered system integration. At the system level, self-functional solutions are constructed through the in-depth integration of sensing units with energy collection modules, such as triboelectric nanogenerators, to create long-term, independently operating intelligent microsystems matching the needs of unattended aerospace monitoring.
5. Standardization and industrialization promotion. Initiative must be taken in establishing unified performance evaluation standards, reliability testing norms, and quality certification systems. The construction of clinical approval channels for medical devices for the healthcare monitoring scenario must be promoted, laying the foundation for the clinical transformation and commercial application of La_1-x_Sr_x_MnO_3_-based flexible sensors. Efforts should be concentrated on formulating special accelerated aging test specifications and performance failure threshold standards for LSMO-based multi-modal flexible sensors. The environmental reliability evaluation system under multi-stress coupling conditions (high temperature and humidity, salt spray, and cyclic bending) should be improved, and a standardized test system that supports the clinical registration of medical wearable devices should be developed.

## 7. Conclusions

As a significant branch of flexible electronics technology, La_1-x_Sr_x_MnO_3_-based flexible wearable sensors are driving the evolution of human–machine interaction from single-command control to multi-dimensional perception collaboration. This is attributed to their unique multimodal sensing capabilities (e.g., temperature, strain, and magnetic field), operational stability across a wide temperature range (20–773 K) and excellent mechanical flexibility. Through the collaborative innovation in material doping modification (e.g., A-/B-site element substitution), process optimization (e.g., nanostructure design and roll-to-roll printing), and multi-functional integration strategies, the comprehensive performance of La_1-x_Sr_x_MnO_3_ sensors has consistently improved, and their functional scope has continuously expanded. Currently, this material has demonstrated a wide-ranging application foundation in fields such as magnetic storage, conductive electrodes, and electrochemical devices.

Looking forward, through the rational design of novel perovskite heterojunctions, the integration of lightweight artificial intelligence algorithms onto chips, and the development of environmentally-friendly electronic packaging processes, La_1-x_Sr_x_MnO_3_-based flexible sensors are expected to play a more critical role in cutting-edge fields such as personalized medicine, smart Internet of Things (IoT), and extreme environment detection, providing important technical support for establishment of an intelligent society deeply integrated with humans, machines, and the environment.

## Figures and Tables

**Table 1:** Main physical properties of La_1-x_Sr_x_MnO_3_ materials.

Performance Parameters	Numerical Ranges	Influencing Factors	Application Correlations
Temperature Coefficient of Resistivity	−(1–5)%/K	Sr Doping Concentration, Oxygen Vacancies	Temperature Sensitivity
Magnetoresistance Ratio	A maximum value of up to 100%	Magnetic Field Strength, Temperature	Magnetic Sensing Capability
Bending Durability	>3600 cycles	Substrate Material, Film Thickness	Mechanical Stability
Operating Temperature Range	20–773 K	Material Composition, Interfacial Bonding	Applicability in Extreme Environments
Bending Endurance	Millisecond-scale	Film Quality, Interfacial Thermal Conductivity	Dynamic Monitoring Capability

**Table 2:** Comparison of the main manufacturing processes of LSMO-based sensors.

Preparation Methods	Process Characteristics	Advantages	Limitations	Applicable Scenarios	Suitability for High-Sensitivity Devices	Suitability for Wearable Mass Production
Interface Engineering	Regulate substrate surface hydrophobicity, chemical modification, and microstructure	Improve film formation quality, enhance interfacial bonding force, reduce delamination risk	High requirement for surface treatment uniformity, specific process compatibility	High-performance implantable sensors, spin electronic magnetic sensor	High (Eliminates interface-related signal drift)	Low (Poor batch consistency)
Transfer Technology	High-quality growth on rigid substrates followed by transfer to flexible substrates via adhesive force	High crystalline quality of films, retention of intrinsic properties, compatibility with various substrates	Complex process, yield needs improvement	High-performance flexible electronics, heterogeneous integrated devices	Very High (Retains intrinsic material properties)	Medium (Limited by large-area transfer yield)
Laser-induced Processing Technology	Femtosecond laser direct-writing, non-contact patterning, programmable control	High precision, simplified process, maskless, supports rapid prototyping and customization	High equipment cost, efficiency of large-area processing is limited	High-precision micro–nano sensors, patterning of flexible electrodes	High (Enables micro–nano patterning without performance degradation)	Low (Low throughput, high equipment cost)
Encapsulation Process	Encapsulation and insulation protection using flexible materials like PDMS	Good biocompatibility, high flexibility, strong fatigue resistance, can conform to complex curved surfaces	Precise control of encapsulation thickness and adhesion is required, long-term hermeticity needs validation	Wearable devices, biomedical implants, flexible e-skins	Medium (Protects against interference but adds minor stress)	Very High (Mature, scalable process)

**Table 3:** Comparison of LSMO with representative flexible sensing materials.

Material System	Key Response	Cycling Stability	Operating Temperature Range	Cost/Fabrication Complexity	Demonstrated Applications	References
LSMO	Strain sensitivity: ΔR/R_0_ ≈ 5%, bending radius 3 mm. Magnetoresistance (MR): 32%(20 K, 2 T)	3600 bending cycles, ΔR/R_0_ degradation < 0.5%	−253–500 °C	High (pulsed laser deposition)	Aerospace, harsh-environment flexible electronics, multimodal sensing	[6]
Graphene	Strain gauge factor (GF):2175.8 Thermal index: 12,015.86 K (25–300 °C)	1000 stretching cycles (0–5% strain)	–40–300 °C	Low (solvent evaporation)	Harsh-environment-flexible electronics; Human joint motion monitoring, respiration detection	[48]
MWCNT	Temperature TCR: −1.18 × 10^−3^/°C (30–300 °C) Bending sensing: 0–88°	3000 bending cycles	30–600 °C	Low (dispensing printing)	Firefighting suits, petroleum industry	[49]
MXene	Piezoresistive sensitivity: 80 kPa^−1^ (−5 °C) 156 kPa^−1^ (RT) 20 kPa^−1^ (150 °C)	10,000 cycles (RT), 2000 cycles (100 °C), 500 cycles (–5 °C:)	–5–150 °C	Medium (electrospinning and dip-coating)	Wearable health monitoring (pulse and joint motion), human–machine interaction	[50]

**Table 4:** Summary of research progress and core gaps in biosafety and environmental tolerance of La_1-x_Sr_x_MnO_3_ based flexible wearable sensors.

Evaluation Dimension	Core Test Items	Existing Research Progress	Standard Basis	Core Research Gaps
Long-term Biocompatibility	In vitro cytotoxicity	24–72 h leach liquor, cell viability > 80% (non-cytotoxic)	ISO 10993-5	Lack of >12 months long-term cytotoxicity data; no bending-coupled toxicity test
	Ion release kinetics	28 d cumulative release: Sr^2+^ 0.51 μg/cm^2^, Mn 0.38 μg/cm^2^	ISO 3160	Lack of long-term release data; no study on sweat composition/temperature fluctuation effects
Environmental Tolerance	85 °C/85%RH aging	1000 h aging, PDMS swelling rate 2.7%, peel strength down 42%	IEC 60068-2-78 [86]	Lack of life extrapolation data; no multi-stress coupled aging test
	Salt spray aging	500 h aging, device resistance drift > 20%	IEC 60068-2-11 [87]	Lack of special aging specifications and failure threshold for LSMO sensors

## Data Availability

The original contributions presented in this study are included in the article. Further inquiries can be directed to the corresponding authors.
